# Demethylation of the *MIR145* promoter suppresses migration and invasion in breast cancer

**DOI:** 10.18632/oncotarget.18686

**Published:** 2017-06-27

**Authors:** Shui-Yi Liu, Xiao-Yi Li, Wei-Qun Chen, Hui Hu, Bo Luo, Yu-Xiang Shi, Tang-Wei Wu, Yong Li, Qing-Zhi Kong, Hong-Da Lu, Zhong-Xin Lu

**Affiliations:** ^1^ Department of Medical Laboratory, Central Hospital of Wuhan, Wuhan 430014, China; ^2^ Cancer Research Institute of Wuhan, Wuhan 430014, China; ^3^ Department of Central Laboratory, Central Hospital of Wuhan, Wuhan 430014, China; ^4^ Key Laboratory for Molecular Diagnosis of Hubei Province, Central Hospital of Wuhan, Wuhan 430014, China; ^5^ Department of Oncology, Central Hospital of Wuhan, Wuhan 430014, China; ^6^ Department of Pathology, Central Hospital of Wuhan, Wuhan 430014, China; ^7^ Department of Cancer Biology, Lerner Research Institute, Cleveland Clinic, Cleveland, OH 44195, USA

**Keywords:** miR-145, methylation, breast cancer, ANGPT2, migration and invasion

## Abstract

miR-145 has been implicated in the progression of breast cancer. Here, we report that its expression is decreased in breast cancer specimens and cell lines and that this low level of expression is associated with DNA methylation of its gene, *MIR145.* Methylation of *MIR145* has previously been correlated with cell migration and invasion, both *in vivo* and *in vitro*. We found that demethylation of *MIR145* reactivates miR-145 and contributes to the anti-cancer properties of 5-aza-2′-deoxyazacytidine (5-AzaC). Therefore, miR-145 is a potentially valuable biomarker for breast cancer.

## INTRODUCTION

Breast cancer is the most common and lethal malignancy in women, with approximately 1.7 million new cases and more than 0.5 million deaths occurring in 2012 [1]. Like most solid tumors, it is not the primary tumor, but the metastasis to distant sites that is the major cause of mortality in breast cancer patients [2]. Thus, it is imminent to identify molecules that suppress the metastasis of breast cancer cells and serve as novel targets for therapeutic development.

MicroRNAs (miRNAs), which belong to the non-coding RNA family, play vital roles in post-transcriptional gene regulation [3]. Many studies have shown that miRNAs contribute to tumor formation and development, suggesting that they act as oncogenes or tumor suppressors [4–6]. miRNAs play important regulatory roles in various cancer processes, such as cancer cell proliferation, apoptosis, cell migration, and invasion [7–9]. miR-145, transcribed from the miRNA cluster at chromosome 5q32, was found to be a tumor suppressor that is downregulated in many human cancers, such as breast, prostate, bladder, colon, and ovarian [10–14]. However, the underlying mechanism for the miR-145 down-regulation in breast cancer has not been fully elucidated.

Epigenetic regulation involving DNA methylation is closely associated with gene expression, and promoter hypermethylation is a major mechanism that silences tumor-suppressive genes in human cancers [15]. Recently, accumulating evidence has shown that hypermethylation in the miRNA gene promoter region represses miRNAs in several cancers [16–18]. These tumor-suppressive miRNAs repressed by promoter hypermethylation play critical roles in cancer development, including cell proliferation, migration and invasion, apoptosis, and cell cycle arrest. Indeed, methylation-silencing of tumor suppressor miRNAs, including miR-491-5p, miR-149, miR-124a, and miR-375, have been demonstrated in breast cancer [19–22].

In the present study, we found that the promoter region of *MIR145* (encoding miR-145) is hypermethylated in breast cancer clinical samples and cell lines. In addition, the function of *MIR145* promoter methylation in driving metastases in breast cancer was explored. Furthermore, we showed that miR-145 represses migration and invasion in breast cancer cells by directly targeting the angiopoietin 2 gene *(ANGPT2)*.

## RESULTS

### miR-145 is significantly downregulated, and *MIR145* is highly methylated in breast cancer

**Figure 1:**
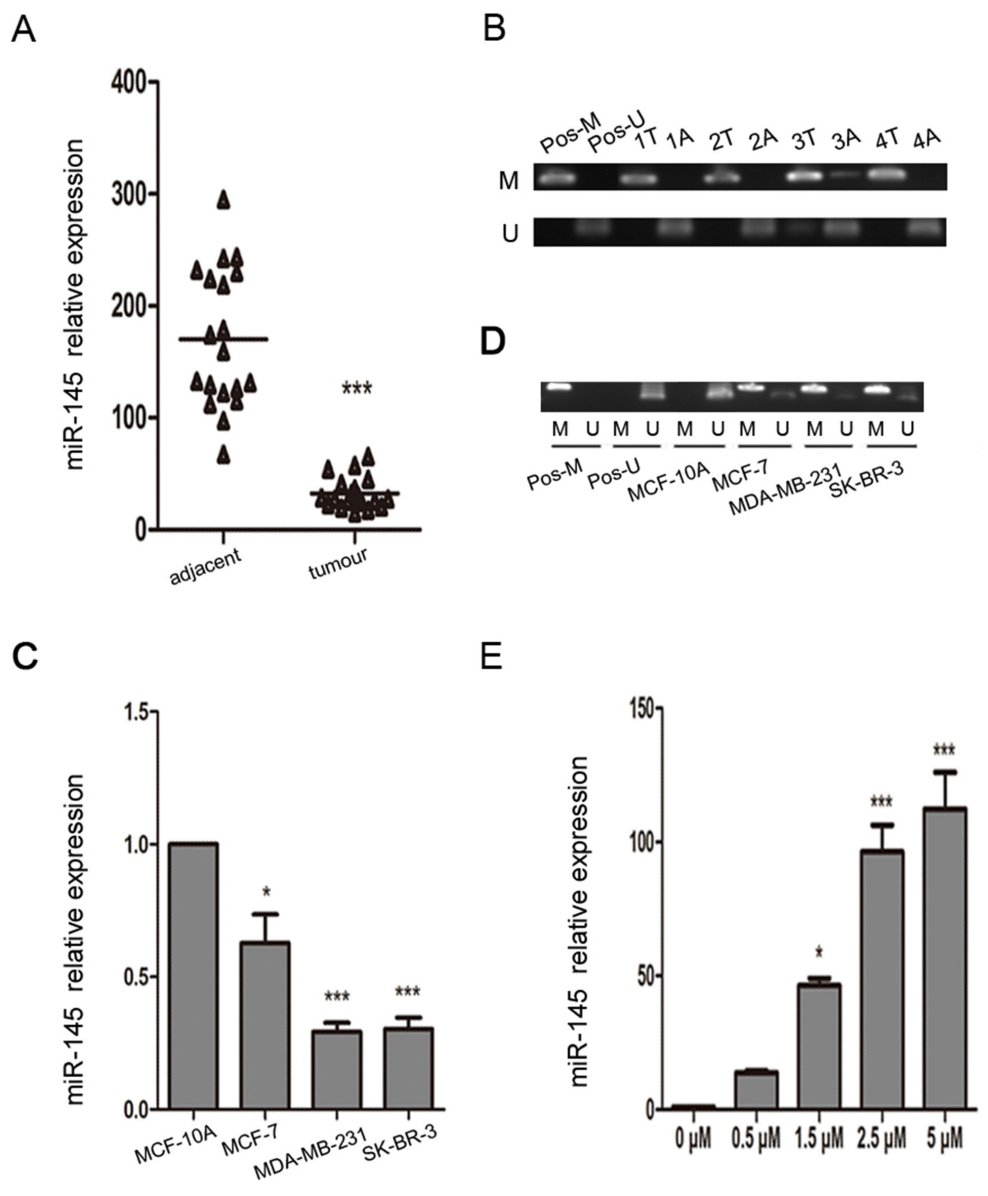
miR-145 is epigenetically downregulated in breast cancer clinical samples and cell lines **(A)** Real-time PCR was used to analyze the expression of miR-145 in 19 breast cancer tissues paired with adjacent noncancerous tissues. **(B)** Methylation-specific PCR (MSP) was used to analyze the methylation state of the *MIR145* promoter region in breast cancer clinical samples determined by MSP. Peripheral blood cell DNA was used as positive control for methylated gene (Pos-M), bisulfite-modified peripheral blood cell DNA as positive control for unmethylated gene (Pos-U), M, methylated gene; U, unmethylated gene; T, tumor tissue; A, adjacent tissue. **(C)** Real-time PCR was used to analyze the expression of miR-145 in breast cancer cell lines. The expression of miR-145 was significantly downregulated in the breast cancer cell lines MCF-7, MDA-MB-231, and SK-BR-3 compared with the normal breast cell line MCF-10A, with U6 as internal control. **(D)** MSP was used to analyze the methylation state of the *MIR145* promoter region in breast cancer cell lines. **(E)** Real-time PCR was used to analyze the expression of miR-145 in MDA-MB-231 cells treated with different concentrations of 5-AzaC (0.5 μM, 1.5 μM, 2.5 μM, and 5 μM). All the assays were performed in triplicates, and the results are shown as the mean ± SD. Significance was determined by Student t-test (A), or one-way ANOVA with Bonferroni’s Multiple Comparison Test (C, E). *P < 0.05, ***P < 0.001.

To determine whether miR-145 is silenced in breast cancer, we examined the expression of miR-145 in 19 breast cancer tissues paired with adjacent normal tissues.We found that miR-145 was significantly downregulated in carcinoma tissues compared with the matched adjacent tissues (Figure 1A). We then examined the expression of miR-145 in three human breast cancer cell lines (MCF-7, MDA-MB-231, and SK-BR-3) and a non-cancerous breast epithelial cell lines MCF-10A. The expression of miR-145 was significantly lower in the breast cancer cell lines (Figure 1C).

As it is reported that miR-145 is downregulated by DNA methylation in other cancers, we hypothesized that DNA methylation is responsible for the downregulation of miR-145 in breast cancer. We performed methylation-specific PCR (MSP) analysis to detect the methylation state of the *MIR145* promoter region. Indeed, breast cancer samples with low expression of miR-145 showed hypermethylation in the *MIR145* promoter (Figure 1B), and hypermethylation of the CpG sites in the *MIR145* promoter was also observed in the MCF-7, MDA-MB-231, and SK-BR-3 cell lines compared with the MCF-10A cell line (Figure 1D). After treatment with different concentrations of the demethylating agent 5-aza-2′-deoxyazacytidine (5-AzaC), the expression of miR-145 was found to be significantly upregulated in a dose-dependent manner in MDA-MB-231 cells (Figure 1E).

### miR-145 targets *ANGPT2 in breast cancer*

**Figure 2:**
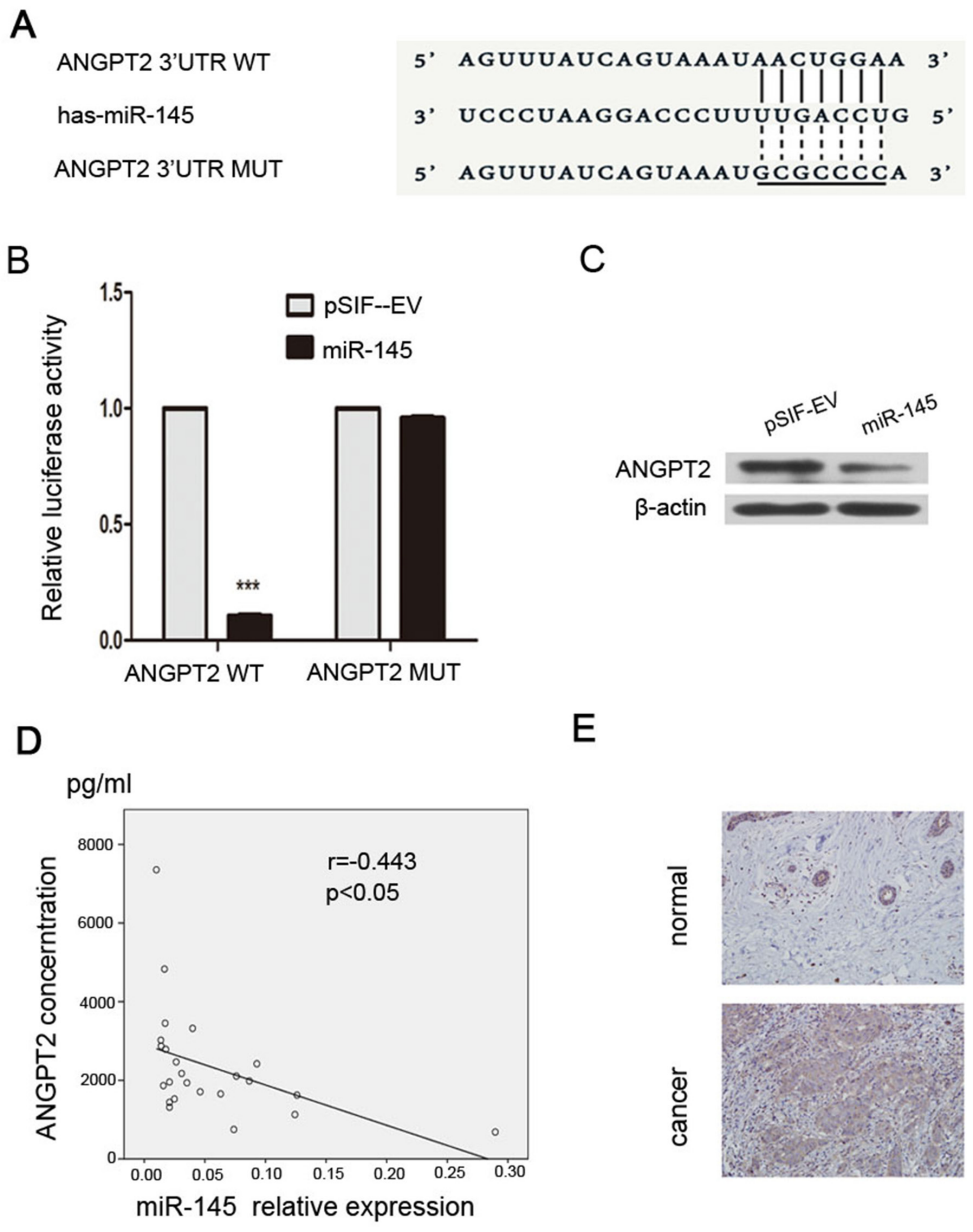
*ANGPT2* is a target gene of miR-145 **(A)** The wildtype and mutant *ANGPT2* 3’-UTR binding site for miR-145. **(B)** Luciferase reporter assay for HEK-293T cells cotransfected with reporter plasmids containing WT or mutant miR-145 binding sites in the ANGPT2 3’-UTR, together with either the pSIF–GFP–miR-145 or pSIF–GFP vectors. **(C)** pSIF–GFP–miR-145 transfection down-regulated the levels of ANGPT2. **(D)** The expression of miR-145 is inversely correlated with ANGPT2 expression in 24 breast cancer samples. qRT-PCR was performed to determine the expression of miR-145 and ELISA was performed to determine the expression of ANGPT2 in the plasma. **(E)** Immunohistochemistry analysis of ANGPT2 expression in adjacent normal breast epithelium and carcinoma (magnification 200×). All the assays were performed in triplicates, and the results are shown as the mean ± SD. Statistical significance was determined by Student t-test. ***P < 0.001.

Using online databases (TargetScan, miRDB, miRanda) we discovered that *ANGPT2* is a potential target gene of miR-145, and the binding sites for miR-145 in the 3’-UTR of *ANGPT2* mRNA are shown in Figure 2A. To validate a direct binding and repression effect, we performed dual-luciferase reporter assays. We found that overexpression of miR-145 significantly reduced the luciferase activity of the wildtype *ANGPT2* 3’-UTR but not that of the mutant *ANGPT2* 3’-UTR in HEK-293T cells (Figure 2B). Moreover, we also found that miR-145 downregulated the expression of ANGPT2 in MDA-MB-231 cells (Figure 2C). To establish a relationship between miR-145 and ANGPT2, we measured the plasma levels of miR-145 and ANGPT2 in breast cancer patients. We observed a statistically significant inversed correlation between miR-145 and ANGPT2 in breast cancer samples (Figure 2D). Next, we found that while ANGPT2 is expressed in at a lower level normal breast epithelium, than it is in breast cancer tissues (Figure 2E). Taken together, our results indicate that ANGPT2 is a direct target of miR-145.

### ANGPT2 is the key mediator of the effects of miR-145

**Figure 3:**
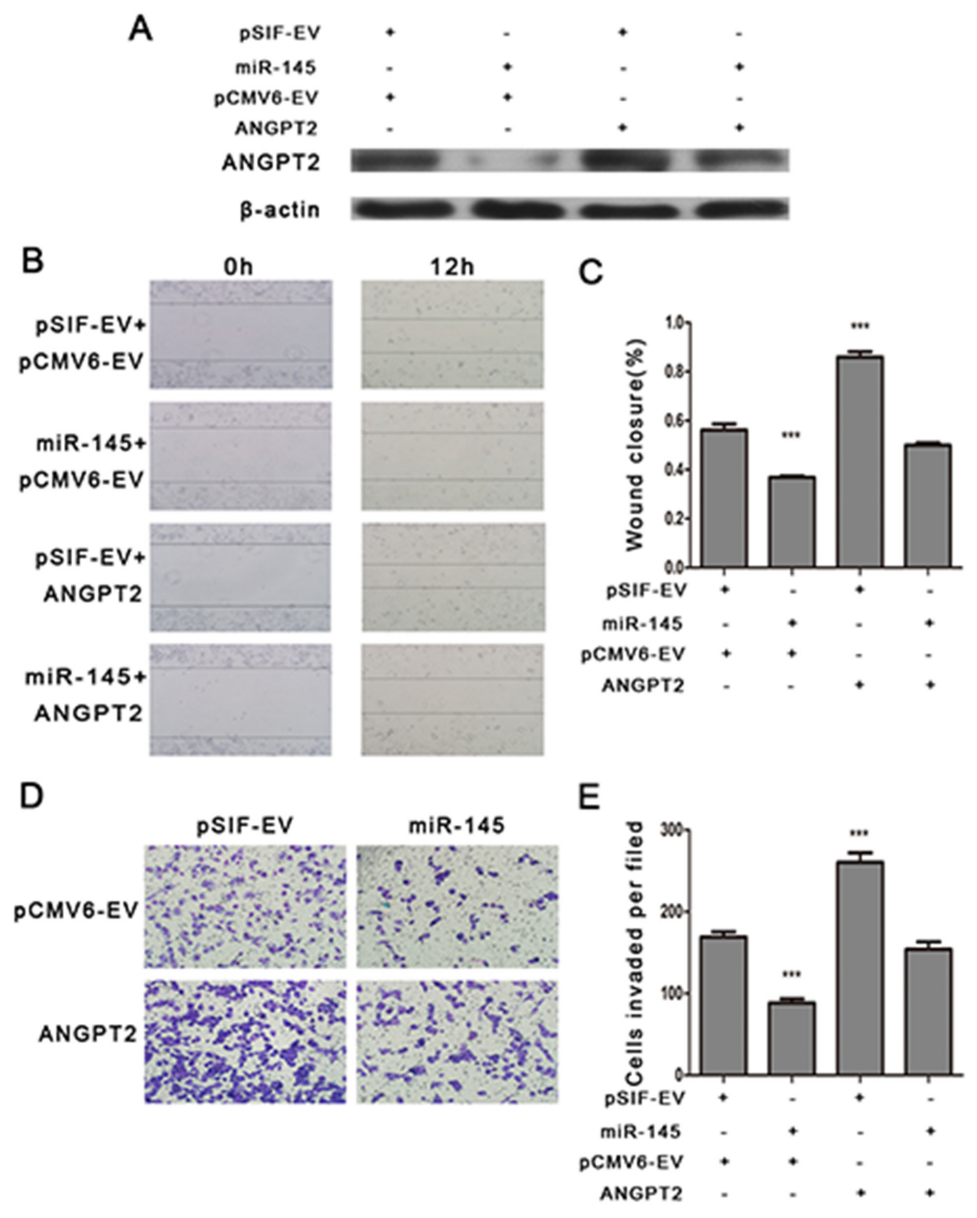
ANGPT2 re-expression attenuates miR-145 mediated inhibition of cell migration and invasion in MDA-MB-231 cells **(A)** Western blot analyzing the expression of ANGPT2 in MDA-MB-231 cells with the co-transfection of pSIF–GFP or pSIF–GFP–miR-145 vectors together with either pCMV6-AC-GFP or pCMV6-AC-ANGPT2 vectors. **(B)** and **(C)** Wound healing assay, **(D)** and **(E)** transwell assays indicated that co-transfection of ANGPT2 with miR-145 profoundly promoted the cell migration and invasion compared with miR-145 transfection alone. All the assays were performed in triplicates, and the results shown as the mean ± SD. Statistical significance was determined by one-way ANOVA with Bonferroni’s Multiple Comparison Test (C, E). ***P < 0.001.

To determine the extent to which ANGPT2 contributes miR-145’s tumor suppression functions, we ectopically expressed an ANGPT2 construct together with miR-145 in MDA-MB-231 cells. Higher expression of ANGPT2 was detected in cells with the ANGPT2 construct (Figure 3A). miR-145 inhibited cell migration and invasion. yet co-transfection of ANGPT2 and miR-145 profoundly reversed them. (Figure 3B–3E). These results showed that ANGPT2 can partially rescue miR-145-suppressed migration and invasion in MDA-MB-231 cells, implicating that ANGPT2 is a functional terget of miR-145 in breast cancer cells.

### Attenuation of the effects of 5-AzaC by knocking down miR-145 *in vitro*

**Figure 4:**
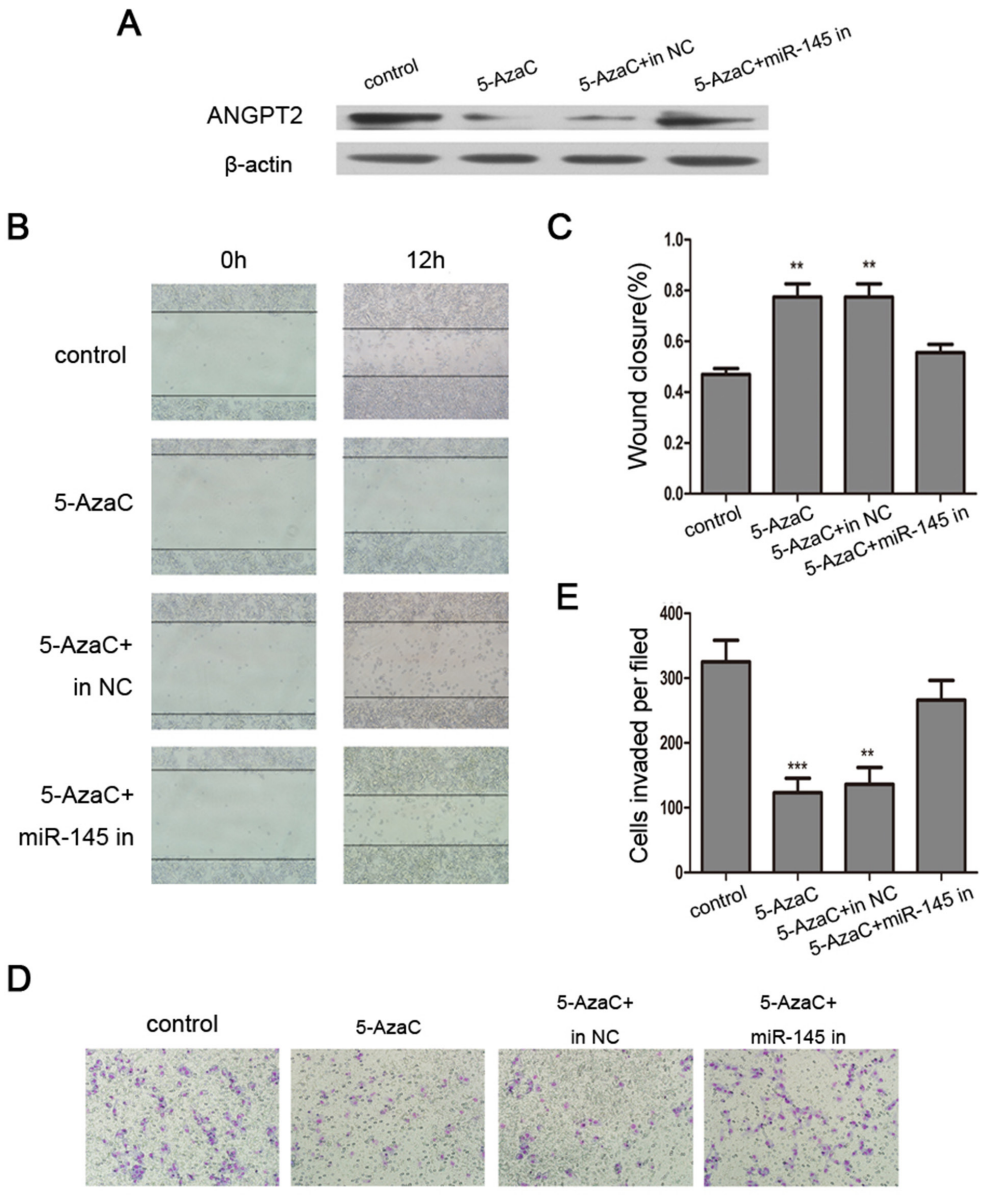
Reversing the effects of 5-AzaC by a miR-145 inhibitor in MDA-MB-231 cells **(A)** Western blot analysis of ANGPT2 indicated that 5-AzaC-evoked downregulation of ANGPT2 protein was reversed by miR-145 inhibitor. **(B)** and **(C)** A wound healing assay indicated that the miR-145 inhibitor reversed the inhibition of cell migration by 5-AzaC (magnification 100×). **(D)** and **(E)** Transwell assays indicated that miR-145 inhibitor reversed the inhibition of cell invasion by 5-AzaC (magnification 200×). All the assays were performed in triplicates, and the results are shown as the mean ± SD. Statistical significance was determined by one-way ANOVA with Bonferroni’s Multiple Comparison Test (C, E). **P < 0.01, ***P < 0.001.

Based on the result that miR-145 directly targets ANGPT2 and is itself silenced by DNA methylation in breast cancer cell lines, we speculated that miRNA-mediated regulation might be involved in 5-AzaC-induced ANGPT2 repression in breast cancer. We transfected 5-AzaC-treated MDA-MB-231 cells with miR-145 inhibitor or an inhibitor control. We found that 5-AzaC-induced downregulation of ANGPT2 protein was strongly reversed by miR-145 inhibitor (Figure 4A). We also found that 5-AzaC treatment inhibited cell migration and invasion of MDA-MB-231 cells, which were partially reversed by co-treatment with the miR-145 inhibitor (Figure 4B–4E).

### 5-AzaC treatment suppresses breast cancer metastasis *in vivo*

**Figure 5:**
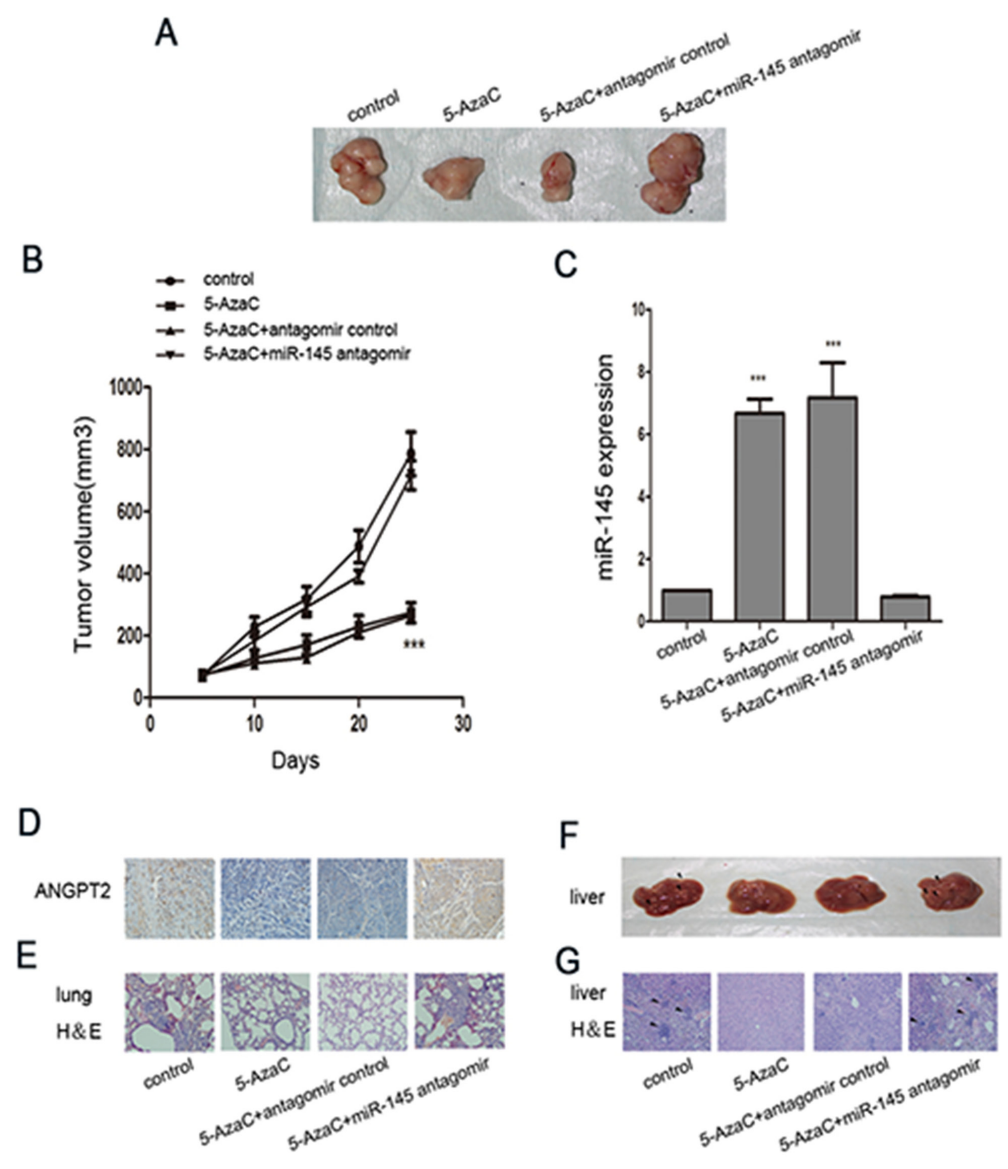
Treatment with 5-AzaC with or without miR-145 antagomir modulated invasion and metastasis in tumor-bearing nude mice **(A)** Representative tumor images in nude mice seeded with MDA-MB-231 cells in the presence of 5-AzaC, 5-AzaC plus miR-145 antagomir, or 5-AzaC plus miR-145 antagomir control (n=6 for each group). **(B)** Time course of tumor growth in these groups. **(C)** The relative expression levels of miR-145 in nodules from each group. **(D)** Immunohistochemistry analysis of ANGPT2 expression in tumors from xenografted mice of the four groups (magnification 200×). **(E)** H&E staining of lungs in the four groups of xenografted mice (magnification 200×). **(F)** Nodules (arrowheads) on liver metastases in the four groups of xenografted mice. **(G)** H&E staining of livers in the four groups of xenografted mice (magnification 200×). All the assays were performed in triplicates, and the results are shown as the mean ± SD. Statistical significance was determined by one-way ANOVA with Bonferroni’s Multiple Comparison Test (B, C). ***P < 0.001.

To determine the *in vivo* effect of 5-AzaC, we used BALB/C nude mice seeded with MDA-MB-231 cells. Figure 5A showed the tumors collected from animals at the end of the experiments. Tumor size and miR-145 expression were significantly decreased in the group of mice treated with 5-AzaC or the combined treatment of 5-AzaC with the antagomir control (Figure 5B, 5C), yet the combined treatment with both 5-AzaC and miR-145 antigomir led tumors to grow at a rate similar to the untreated group. Immunohistochemistry showed that 5-AzaC-evoked downregulation of ANGPT2 protein was markedly enhanced by miR-145 antagomir (Figure 5D). Moreover, lung and liver metastasis was completely inhibited by 5-AzaC, while miR-145 antagomir attenuated the effects of 5-AzaC (Figure 5E–5G).

## DISCUSSION

miRNAs play critical roles in almost every aspect of cancer [23] and often act as oncogenes or tumor suppressors to regulate many cellular events at various steps of tumor formation and progression. Although evidence has highlighted the importance of miR-145 as a tumor suppressor in breast cancer, the precise molecular mechanisms remain largely unknown. In this study, we examined the epigenetic state of the miR-145 promoter in breast cancer clinical samples and cell lines and found that the miR-145 promoter was methylated in both. We also showed that the effects of the demethylating agent 5-AzaC were abolished by simultaneous knockdown of miR-145. To better understand the tumor-suppressive effect of miR-145 in breast tumorigenesis, we used online databases to predict the targets of miR-145 and confirmed that ANGPT2 was the direct target of miR-145. We conclude that methylation of miR-145, which silences its expression and results in ANGPT2, upregulation implying an epigenetic contributor to breast tumorigenesis.

ANGPT2 is a member of the angiopoietin family, which contributes to tumor development and progression by linking the metastatic inflammasome with the angiogenic program. It is reported that ANGPT2 is an important molecular determinant of cancer cell metastasis [24–26]. An increasing body of evidence has shown that ANGPT2 is associated with tumor angiogenesis and progression in liver cancer [27], lung cancer [28], pancreatic cancer [29], and breast cancer [25, 30]. Circulating ANGPT2, as a biomarker of disease status, is associated with patient prognosis in multiple cancers [31–33]. As circulating miRNAs are informative biomarkers for cancer diagnosis and prognosis [34, 35], we measured circulating miR-145 and ANGPT2 levels in both breast cancer patients and healthy controls. The levels of miR-145 were decreased and ANGPT2 levels were increased in breast cancer patients compared with healthy controls. These results support the possible utility of circulating miR-145 in combination with ANGPT2 as a biomarker for tumor development and prognosis.

Recently, it is shown that miRNA genes are epigenetically modified in various cancers. DNA methylation-mediated downregulation of miR-145 is found in prostate [36], ovarian [37], and lung cancer [38]. In this study, we found that miR-145 expression has a significant inverse correlation with its promoter methylation level in breast cancer cell lines. 5-AzaC, a methylation inhibitor used to restore the expression of miR-145 in MDA-MB-231 cells, reduced migration and invasion *in vitro* and metastasis in an animal model. To our knowledge, this is the first report of an association between low miR-145 expression and DNA methylation in breast cancer. Based on our findings, demethylation that reactivates the tumor suppressor miR-145 contributes, at least partially, to the anti-cancer properties of 5-AzaC. This miRNA, therefore, is a suitable epigenetic target for cancer therapeutic development.

In conclusion, the present study shows that the reduced expression of miR-145 in breast cancer is due to DNA methylation, and miR-145 represses cell migration and invasion by targeting ANGPT2 both *in vivo* and *in vitro*. miR-145 promoter methylation state and expression levels are valuable biomarkers in breast cancer, and further investigation is underway to develop these biomarkers for clinical use.

## MATERIALS AND METHODS

### Clinical samples

Human breast cancer tissues and matched normal adjacent breast tissues were obtained from the Central Hospital of Wuhan in 2014. None of the patients received radiotherapy or chemotherapy before the operation. Informed consent was obtained from all patients before sample collection and this study was approved by the Medical Ethics Committee of the Central Hospital of Wuhan, and all human subject research was performed in accordance with institutional and national guidelines, as well as Declaration of Helsinki requirements. The samples were fresh-frozen and stored in liquid nitrogen after surgery. Preoperative 2-ml blood samples from the patient and control groups were collected in EDTA-containing tubes and processed within 4 hours. Plasma samples were centrifuged at 1000 x g for 15 min at 4°C, and the supernatants were stored in RNase-free tubes at –80°C until measurement.

### Cell culture

HEK-293T, MCF-10A, MCF-7, MDA-MB-231, and SK-BR-3 cell lines were purchased from the American Type Culture Collection (ATCC). MCF-10A and MCF-7 cells were cultured in RPMI-1640 medium; HEK-293T and MDA-MB-231 cells were cultured in Dulbecco’s modified Eagle’s medium (DMEM); and SK-BR-3 cells were cultured in McCoy’s 5A medium. All media were supplemented with 10% fetal bovine serum (FBS), 100 U/ml penicillin, and 100 μg/ml streptomycin. All cells were grown at 37°C in a humidified atmosphere of 95% air and 5% CO_2_.

### Quantitative real-time PCR

Plasma miRNA was extracted using the mirVana PARIS kit (Ambion, Carlsbad, CA, USA) according to the manufacturer’s instructions. Total RNA from cultured cells and tissue samples was extracted using TRIzol (Invitrogen, Carlsbad, CA, USA). Quantitative real-time reverse transcription (qRT)-PCR was conducted using the TaqMan miRNA Reverse Transcription kit, the TaqMan Small RNA Assays kit, and TaqMan Universal PCR Master Mix (Applied BioSystems, Carlsbad, CA, USA). To normalize the data for quantification of miRNAs, U6 was selected as the endogenous control. miRNA expression levels were determined using the ΔΔCt method.

### DNA extraction and methylation-specific PCR (MSP)

Genomic DNA was extracted from breast cancer cell lines using the QIAamp DNA Mini Kit (Qiagen, Hilden, Germany) according to the protocol. The methylation state of CpG sites in the *MIR145* promoter was determined by the bisulfite sequencing method, as described by Clark *et al.* [39], although the method was modified as follows: DNA (1 μg) was diluted in 50 μl double-distilled water, to which was added 5.5 μl of 3 M NaOH, and the sample was incubated at 55°C for 20 min. Hydroquinone (30 μl of 10 mM) and 520 μl of 3.6 M NaHSO_3_ (pH 5.0) were added to the denatured DNA solution, and the tube was incubated at 55°C for 16 h. The NaHSO_3_-treated DNA was then purified using the Wizard DNA Clean-up System (Promega, Madison, WI, USA), denatured with 3 M NaOH, precipitated with ethanol, and dissolved in 20 μl double-distilled water. The methylation state of the *MIR145* promoter region was determined by MSP using bisulfite-modified DNA as described by Suh et al. [40]. The primers for the Unmethylated form of miR-145 were 5’- GGG TTT TTG GTA TTT TTT AGG GTA ATT GAA GTT TT -3’ (sense), 5’- AAC CAA AAT AAA ATA CCA CAC ATC ACC A-3’ (antisense). The primers for the methylated form of miR-145 were 5’-GGG TTT TCG GTA TTT TTT AGG GTA ATT GAA GTT TC-3’ (sense), 5’- TAA AAT ACC ACA CGT CGC CG-3’ (antisense). The annealing temperature was 64°C for Methylated-PCR, and 56°C for Unmethylated-PCR, with 40 cycles used for each. The CpG methyltransferase (Sss I)-treated and untreated peripheral blood cell DNA from healthy adults were used as methylated and unmethylated positive controls, respectively.

### 5-Aza-2’-deoxyazacytidine treatment

To block DNA methylation, MDA-MB-231 cells were treated with 2.5 μmol/l 5-aza-2′-deoxyazacytidine (5-AzaC; Sigma-Aldrich, MO, USA) for 3 days, during which time the culture medium was replaced every 24 h. Cells were harvested for western blotting, wound healing assays, and Transwell migration assays.

### Wound healing assay

MDA-MB-231 cells were seeded in 6-well plates after transfection or 5-AzaC treatment. After the cells were grown to 80% confluence, an artificial wound was created using a sterile 200-μl pipet tip. The cells were then washed twice with PBS and incubated in medium with 1% FBS at 37 °C. The wound-healing images were taken 12 h later.

### Transwell migration assay

Cell invasion was assessed using Transwell invasion chambers coated with Matrigel. Cells (5×10^4^) suspended in 200 μl of serum-free medium were seeded into a Matrigel invasion chamber. The bottom chamber was filled with 500 μl of DMEM with 10% FBS. After 24 hours, the non-invading cells were removed with cotton swabs. Invasive cells, located on the lower surface of the chamber, were stained with Giemsa and counted.

### Dual-luciferase reporter assay

miR-145 binding sites in the *ANGPT2* 3’-UTR were amplified by PCR (sense, 5’- TAT CTA GAC TGA CGG GAC CCA CAT GC -3’; antisense, 5’- ATG CGG CCG CTT GCC TAC CAA TGT ACT G -3’). The mutated 3’-UTR of *ANGPT2* was also amplified (sense, 5’- TAT CTA GAA TCA GTA AAT GCG CCC CAA A -3’; antisense, 5’- ATG CGG CCG CTT GCC TAC CAA TGT ACT G -3’). Both wild-type and mutant PCR products were cloned downstream of the reporter gene between the XbaI and NotI sites of the pRL-TK vector (Promega). The pGL3 Vector was used as the control. As previously described [41], HEK-293T cells were cotransfected with either the pRL-TK–ANGPT2-3’UTR or pRL-TK–ANGPT2-3’UTR mutant vectors (100 ng) together with 10 ng of the pGL3 control (Promega) and 60 ng of pSIF–GFP–miR-145 precursor plasmid using Lipofectamine® LTX and Plus reagents (Invitrogen) according to the manufacturer’s protocol. Luciferase activities were then measured 48 h after transfection using a Dual-Glo luciferase reporter assay system (Promega).

### Western blot

MDA-MB-231 cells were washed twice with cold PBS, and protein was extracted using the RIPA buffer. The protein concentration was then determined using the BCA protein assay kit (Pierce, Rockford, IL, USA) and separated by 10% SDS-PAGE. A rabbit ANGPT2 primary antibody (1:1000, Sigma-Aldrich) was used, and an anti-β-actin (1:1000, Santa Cruz, Delaware, CA, USA) antibody was used to normalize the amount of the analyzed samples. Labeled bands were detected using ECL Select western blotting detection reagent (GE Healthcare, Buckinghamshire, UK).

### Enzyme-linked immunosorbent assay (ELISA)

Plasma ANGPT2 levels were measured with the Quantikine ELISA Human Angiopoietin-2 Immunoassay kit (R&D Systems, Minneapolis, USA).

### Xenograft assays

Female BALB/C nude mice (4–6 weeks old) were purchased from Beijing HFK Bio-Technology (Beijing, China). The animals were housed in a specific pathogen-free facility at Tongji Medical College (Wuhan, China). All the animal experiments were conducted under a protocol approved by the Institutional Animal Care and Use Committee at Tongji Medical College. MDA-MB-231 cells (1 × 10^7^) in 0.1 ml OptiMEM were injected into the mammary fat pad. Tumor volumes were measured by using a caliper and estimation by the formula: V=D×d^2^×0.5 (V, volume; D, longer diameter; d, shorter diameter). After the tumor volumes approached 60 mm^3^, the nude mice were randomly assigned to four groups (each containing four mice): the control group, the 5-AzaC group, the 5-AzaC-plus-antagomir-control group, and the 5-AzaC-plus-miR-145-antagomir group. The miR-145 antagomir or the control were intratumorally injected three times per week for 2 weeks as described previously ^41^. 5-AzaC (1 mg/kg) was intraperitoneally injected every 2 days for 2 weeks. Three weeks after the first injection, the mice were sacrificed and the tumors, liver, and lungs resected. The tumors were then divided into two sections, one for detection of miR-145 expression by qRT-PCR. The other section and the liver and lungs were fixed in 10%-buffered formalin and embedded in paraffin for immunohistochemistry or hematoxylin & eosin (H&E) staining.

### Statistical analysis

Continuous variables were compared using Student’s t-test or an ANOVA test using Graphpad Prism 5.0 software. The P-values were calculated and are presented in the figures (*P < 0.05, **P < 0.01, and ***P < 0.001).
